# The impact of continuous calorie restriction and fasting on cognition in adults without eating disorders

**DOI:** 10.1093/nutrit/nuad170

**Published:** 2024-01-23

**Authors:** John O’Leary, Chloé Georgeaux-Healy, Lucy Serpell

**Affiliations:** North East London NHS Foundation Trust, CEME Centre, West Wing Marsh Way, Rainham, Essex RM13 8GQ, UK; Research Department of Clinical, Educational and Health Psychology, University College London, London WC1E 6BT, UK; North East London NHS Foundation Trust, CEME Centre, West Wing Marsh Way, Rainham, Essex RM13 8GQ, UK; Research Department of Clinical, Educational and Health Psychology, University College London, London WC1E 6BT, UK

**Keywords:** calorie restriction, cognitive function, eating disorders, fasting, intermittent fasting

## Abstract

Research into the effects of calorie restriction continues to intrigue those interested in whether it may allow humans to live longer and healthier lives. Animal studies of continuous calorie restriction (CCR) and fasting have demonstrated substantial advantages to health and longevity. However, concerns remain about the impact of restricting calorie intake on human health and cognition. Given the emerging evidence of cognitive impairments in eating disorders, studies investigating restricted calorie intake in healthy humans (in an ethical way) may also have implications for understanding restrictive eating disorders. In this review, the published literature on the impact of CCR and fasting on cognitive function in healthy human participants is synthesized. Of the 33 studies of CCR and fasting in humans identified, 23 demonstrated significant changes in cognition. Despite variation across the cognitive domains, results suggest CCR benefits inhibition, processing speed, and working memory, but may lead to impairments in cognitive flexibility. The results of fasting studies suggest fasting is associated with impairments in cognitive flexibility and psychomotor abilities. Overall, the results of these studies suggest the degree (ie, the severity) of calorie restriction is what most likely predicts cognitive improvements as opposed to impairments. For individuals engaging in sustained restriction, this may have serious, irreversible consequences. However, there are mixed findings regarding the impact of CCR and fasting on this aspect of human functioning, suggesting further research is required to understand the costs and benefits of different types of calorie restriction.

## INTRODUCTION

Researchers interested in the impact of continuous calorie restriction (CCR) and fasting diets on health have reported several potential physical health benefits in animals and, more recently, for humans. Thus, there is growing support for several dieting regimes, with advocates of CCR and fasting claiming that following such regimes may yield health benefits such as increased lifespan and protection from dementia and Alzheimer’s disease.^1^ However, there is less research on the cognitive impacts of these diets, as well as a dearth of research that directly compares the effects of CCR and fasting on cognition.

We found only 3 studies that compared the impact of CCR and fasting on cognition in humans.^2–4^ Although the findings from these studies suggest no significant differences between the impact of CCR and that of fasting on cognition, more research is needed directly comparing the 2 diets. We attempt to address this knowledge gap by examining studies that investigated cognition in participants who subscribed to CCR diets, as well as those engaging in fasting. We investigate differences in psychomotor speed, set-shifting capabilities, and mental flexibility; working and prospective memory capacity; and reflective impulsivity. The potential benefits and detriments of CCR and fasting to cognition are discussed, as well as potential implications for restrictive eating disorders (EDs).

### Continuous calorie restriction and its health benefits

CCR refers to intentional reduction in energy intake, often for the purpose of weight loss. Those who follow a CCR regime typically reduce their daily energy intake by between 15% and 30%. CCR has been studied with growing interest because limitation of food intake while maintaining optimum nutrition has been shown to extend lifespan and improve general health in a variety of organisms.^1^ McCay et al^5^ were the first to show that healthy rodents who had their daily energy intake reduced lived longer when compared with a control group fed ad libitum. Numerous experimental studies have confirmed this observation in insect^6^^,^^7^ and mammal species.^8^^,^^9^ Although the exact mechanisms are not yet fully elucidated, these findings have captured the attention of those interested in similar effects within human populations. The recent Comprehensive Assessment of Long-Term Effects of Reducing Intake of Energy (CALERIE) study of short-term sustained CCR in humans reported beneficial changes in body composition and weight, cardiovascular disease, and glucoregulatory function.^10–15^ The CALERIE researchers tested the effects of CCR on humans with both overweight and healthy weight for up to 24 months, safely inducing CCR without malnutrition.^16^ The study showed physiological, psychological, and behavioral improvements.^17^

Interest in the benefits of CCR, particularly weight loss, extends beyond the realms of the scientific community as public awareness about its benefits continue to grow steadily. Obesity is a global epidemic, with 13% of the world’s adult population remaining obese.^18^ Despite government recommendations for CCR to curb obesity, multiple studies have shown that weight loss diets are often ineffective.^19^^,^^20^ Weight lost in a range of diets is frequently modest and often regained once CCR ceases, leaving dieters heavier than when they started.^21^ Therefore, alternative weight loss strategies are of great interest. One such example is fasting.

### Fasting and its health benefits

Fasting refers to the deliberate abstinence from food, drink, or both for health, religious, or other reasons. A common form of fasting is intermittent fasting (IF), which describes a cycle of eating normally for a time, followed by abstaining from food entirely or reduced intake for another period. IF may include alternate-day fasting (ie, fasting every other day), time-restricted feeding (fasting within a specified time each day; eg, Ramadan fasting), or whole-day fasting (typically 1–2 days of complete fasting per week^22^). One example of IF is the 5:2 diet, whereby individuals eat normally for 5 consecutive days and then reduce their energy intake substantially during the remaining 2 days. For the purposes of this review, we will refer to fasting generally, to include IF, alternate-day fasting, time-restricted feeding, and other fasting methods.

Several studies suggest there are benefits to following fasting regimes. In animals, alternate-day fasting is associated with a reduction in body fat,^23^ heart rate, and blood pressure.^24^ These benefits appear to be specific to IF and not just calorie restriction (CR). Indeed, rodents fed following an IF regime with a 15%–30% reduction in intake had greater improvements than those subjected to a 40% daily calorie-restricted diet.^24^ Other rodent studies suggest that fasting can reduce insulin resistance,^25^ reduce the risk of cardiovascular disease,^26^ delay cancer progression,^27^ and prevent neurodegeneration in the brain.^28^

Initial reports in rodents suggest that IF is at least as effective as CCR diets for health benefits such as weight reduction and improved insulin sensitivity.^25^ However, evidence supporting the health benefits of fasting for humans is limited to a handful of randomized controlled trials and observational studies,^29^ most with modest sample sizes and limited duration.^30^ Two recent reviews of IF and its benefits to humans found that most studies concentrated on weight loss and obesity-related health outcomes, with little attention to secondary outcomes such as cognitive performance.^31^^,^^32^

### Continuous calorie restriction, fasting, and cognition

Given possible health benefits of the restriction of calories in humans, questions have arisen regarding the potential impact of CCR and fasting on the brain. One theory described by Mattson^33^ explores the possibility that the human brain has evolved to provide an advantage over competitors during periods when food is scarce, by ensuring optimal brain functionality when searching for nutrients. This suggests that fasting and CCR may have positive effects on brain function. However, given the high glucose requirements of executive function, it is possible that CCR may also be detrimental to some areas of cognitive function. Cognitive function describes numerous mental processes that assist our ability to gain knowledge and comprehension. It allows humans to perceive, reason, store, and manipulate information, and to solve problems based on the information available to them.

Some research suggests that CCR negatively impairs attentional processes^34^ as well as cognitive flexibility,^35^ but other work suggests that CCR may reduce age-related cognitive decline.^36^ Overall, there is a lack of reviews concerning the short-term impact of CCR on cognition in humans. A recent review confirmed this limited evidence,^37^ and another indicated that short-term fasting was associated with cognitive deficits, particularly higher-order functions (eg, attention, flexibility^38^). It is worth noting that these reviews underline the need for more longitudinal studies of fasting, and that, given methodological inconsistencies and shortcomings of current studies, the impact of fasting on cognition remains largely unknown. Moreover, many reviews examining CCR, fasting, and cognition do so separately, or the authors limit their search to studies including another variable (eg, exercise, glucose metabolism, age-related disease).

### Continuous calorie restriction, fasting, cognition, and eating disorders

Understanding which regions of cognition are sensitive to changes in calorie intake in humans may help prevent and treat cognitive impairments and provide insight into eating pathology. For instance, there is emerging evidence for a specific cognitive profile that matches those with EDs. In anorexia (AN), studies have highlighted executive dysfunction in areas relating to response inhibition, decision-making, memory, and cognitive flexibility,^39–41^ with frontal lobe function, particularly set-shifting, frequently identified.^42^ These areas of dysfunction may have implications in the long term. For example, a recent study found an association between AN duration and cognitive impairment,^43^ suggesting that poor cognitive performance may play a role in disorder persistence. Weight-restored patients still perform more poorly than control study participants on cognitive flexibility and memory tasks,^44^ which also highlights potentially scarring effects of chronic malnutrition on cognitive performance.

There is some evidence that the cognitive deficits (eg, impaired set-shifting) observed in those with EDs may also be present in healthy individuals who restrict their food intake during short-term fasting.^45^^,^^46^ Inducing CCR or fasting conditions in individuals with EDs is not ethically appropriate, but exploring the impact of CCR and fasting on various cognitive domains in healthy individuals may improve understanding of cognitive function in those with EDs.

### Aims

This review aims to synthesize the literature on the impact of CCR and fasting on cognitive function in humans without EDs. By directly comparing the impact of these regimes on cognition, we hope to elucidate the possible differences between them. This may better inform researchers who are interested in the prevention and treatment of cognitive impairments as well as those interested in the costs and benefits of calorie restriction. The results of this review also provide information that may shed light on fasting and malnutrition in EDs.

## METHODS

A PsycINFO database search was conducted during October 2018 and updated in January 2023. In the original review, database searching was completed from the earliest entry to October 2018; in the updated review, the literature was searched from October 2018 to January 2023 inclusive. Manual searches were also conducted using Google Scholar. Search terms were discussed with the principal investigator, L.S., and taken from previous literature. These included the following: “5*2 diet,” “2 day fast*,” “alternate day fasting,” “calorie restriction,” “intermittent fast,” “cognition,” “executive funct*,” “decision making,” and “set-shifting,” among others. The search terms were used in various combinations to find articles of interest (see Table 1 for a full list of terms).

**Table 1:** List of terms used for database search

“5*2 diet”	“executive funct*”
“2 day fast*”	“decision making”
“alternate day fasting”	“set-shifting”
“calorie restriction”	“reflective impulsivity”
“periodic fast*”	“psychomotor”
“intermittent energy restriction”	“working memory”
“Fast*”	“prospective memory”
“fasted”	“Trail making”
“intermittent fast*”	“perception”
“intermittent diet”	“visuospatial memory”
“2-day fast*”	“neuropsy*”
“alternate day fasting”	“neuro*”
“periodic fast*”	“visuospatial”
“intermittent energy restriction”	“memory”
“low-calorie”	“brain”
“starvation”	“attention”
“calorie restraint”	“central executive”
“diet* restraint”	“inhibition”
“restricted eating”	“processing”
“cognition”	

We included only experimental studies that measured cognition after the manipulation of total energy intake in human adult participants (aged ≥18 years) participants. We included experimental studies that examined CCR, fasting, or both. We defined CCR as a deliberate reduction in energy intake below 100% (ie, studies in which food intake was reduced but some food was still allowed). We found studies with CCR between 17%–80%. We defined fasting as a period of 100% reduction in energy intake (ie, studies in which there was no intake of calorie-containing foods and drinks) for at least 4 hours. Accordingly, the studies included IF, alternate-day fasting, time-restricted feeding, and other fasting methods. Most of the studies included allowed ad libitum water, although we did not have a particular criterion in our search for this.

The areas of cognition studied were attention, inhibition, set shifting, processing speed, working memory (WM), and psychomotor speed. Attention refers to the cognitive process that allows us to actively process certain stimuli, and inhibition is a cognitive control mechanism that allows us to suppress irrelevant stimuli. Set shifting is a type of cognitive flexibility and refers to the ability to shift attention from 1 task to another. Processing speed refers to the speed at which one can perceive and process information. WM is a part of short-term memory that stores information temporarily. Finally, psychomotor speed refers to the time between cognitive processing and physical response.

Our exclusion criteria were as follows: the use of exercise as an intervention that varied between control and restriction groups, comparing specific dietary manipulations (eg, low-carbohydrate, high-fat diet; Mediterranean diet), unpublished work (eg, dissertation studies), participants with a diagnosed ED, and quasi-experimental methods (eg, observations, interventions).

The initial search yielded a total of 936 results, which was reduced to 25 after accounting for inclusion and exclusion criteria. The updated search found 15 articles, and these were reduced to 8 after accounting for inclusion and exclusion criteria.

## FINDINGS

Here, we explore studies that assessed fasting, CCR, and/or both. Of those reviewed here, 15 were CCR studies and 16 were fasting studies. Only 3 studies directly compared the effects of CCR and IF with respect to cognition. Of the total of 33 studies, 11 used between-participant designs and the remainder used within-participant designs. The effects of CCR and fasting on all cognitive domains described are summarized in Table 2 (CCR), Table 3 (fasting), and Table 4 (both CCR and fasting).

**Table 2:** Effect of continuous calorie restriction on cognition

Reference	Sample population	Duration (d)	Energy reduction (%)	Attention	Inhibition	Set shifting	Processing speed	Working memory	Psychomotor abilities
Brinkworth et al, 2009	106 adults with overweight	365	28	–	–	–	No change	↑	–
Bryan and Tiggemann, 2001	42 women with overweight	84	20	↑	↑	–	–	No change	–
Buffenstein et al, 2000	9 adults with overweight	30	60	–	–	–	↑	–	–
Cheatham et al, 2009	42 adults with overweight	180	30	No change	–	–	No change	–	–
Halyburton et al, 2007	93 adults with obesity	56	30	–	–	–	↑	↑	–
Kretsch et al, 1997	14 healthy adult women	105	50	No change	–	–	↑	–	–
Leclerc et al, 2020	220 nonobese, healthy participants	730	25	–	–	–	–	↑	–
Makris et al, 2013	47 adults with obesity	168	25	No change	↑	No change	–	–	–
Martin et al, 2007	24 adults with overweight	168	25	No change	–	–	–	No change	–
Pearce et al, 2012	44 adults with obesity	56	30	No change	–	–	No change	No change	–
Siervo et al, 2012	50 adults with obesity	116	40	↑	–	–	–	–	–
Solianik et al, 2018	9 adults with obesity	2	75	–	–	↓	–	↑	No change
Wardle et al, 2000	52 adults with elevated serum cholesterol levels	84	17	↓	–	–	–	–	–
Wing et al, 1995	21 women with overweight	28	80	No change	↑	–	–	–	–
Witte et al, 2009	19 elderly participants with overweight	84	30	No change	–	–	–	No change	–

*Symbols:* ↑, significant improvement; ↓, significant impairment; –, the domain was not measured in the study.

**Table 3:** Effect of fasting on cognition (no food or calorie-containing fluids allowed, water only)

Reference	Sample population	Duration of fasting (h)	Duration of study	Energy reduction (%)	Attention	Inhibition	Set shifting	Processing speed	Working memory	Psychomotor abilities
Anton et al, 2019	10 sedentary adults with overweight	16	4 wk	100	No change	–	–	–	No change	–
Benton and Parker, 1998	80 female students	14	8 d	100	–	–	–	–	No change	–
Bolton et al, 2014	60 healthy adults	16	2 d (1 wk apart)	100	–	–	↓	–	–	–
Chamari et al. (2016)	11 healthy, male, trained cyclists	15–17	2 mo	100	↑	–	–	–	–	–
Crhová and Kapounková, 2020	16 young, healthy adults	120	1 wk	100	↓	–	–	–	↓	–
Doniger et al, 2006	40 university students	16	1 d	100	–	No change	–	↓	–	↓
Green et al, 1995	21 female students	24	5 d (in successive weeks)	100	No change	–	–	–	No change	↓
Green et al, 1997	20 healthy students	4	1 d	100	–	–	–	–	No change	↓
Harder-Lauridsen et al, 2017	10 healthy male participants	14	28 d	100	–	–	–	–	No change	–
Howard et al, 2020	33 female students	20	2 d	100	–	–	↓	–	–	–
Owen et al, 2012	30 university students	12	6 d (6-way crossover design)	100	No change	–	–	–	No change	–
Pender et al, 2014	60 healthy adults	18	2 d	100	–	–	↓	–	–	–
Solianik et al, 2016	9 male amateur weight-lifters	48	1 d (fasting for 48 h before)	100	–	–	↑	–	No change	–
Solianik et al, 2020	11 women with overweight or obesity	48	1 d (fasting for 48 h before)	100	–	–	↓	–	No change	–
Stewart and Samoluk, 1997	32 university students	6	1 d	100	–	No change	–	–	–	–
Tian et al, 2011	18 male athletes	12	4 d (2 during fasting, 2 after fasting)	100	↑	–	–	↑	No change	–

*Symbols:* ↑, significant improvement; ↓, significant impairment; –, the domain was not measured in the study.

**Table 4:** Comparing the effect of continuous calorie restriction and fasting on cognition

Reference	Sample population	Duration (d)	Energy reduction	Attention	Inhibition	Set shifting	Processing speed	Working memory	Psychomotor abilities
Teong et al, 2021	46 women with overweight or obesity	56	70%	↑ in both groups	–	–	↑ in both groups	–	–
Zajac et al, 2021	17 healthy or with overweight female participants	7.5	100% and 75%	No change	–	–	No change	No change	–
Kim et al, 2020	43 individuals, aged 35–75 y, with central obesity	28	100% and 20% (500 kcal deficit)	–	–	–	–	↓ in the fasting group	–

*Symbols:* ↑, significant improvement; ↓, significant impairment; –, the domain was not measured in the study.

### Attention

Most of our daily tasks require us to notice discrete stimuli and often involve the need to sustain our attention or to redirect it where necessary until a task is complete. There are risks associated with poor attention in certain occupations, such as those that involve machinery or medical procedures; hence, understanding the impact of CCR and fasting on this domain is important.

#### Continuous calorie restriction studies

In 7 studies, researchers found no changes in attention after CCR, an increase in attention was reported in 3 studies, and a decrease in attention was reported in 1 study. Individuals with overweight who restricted their calories by 25% over 168 days showed no changes in attention, measured using Conner’s Continuous Performance Test II,^47^ which measures attention, concentration, inattention, and impulsivity.^48^ Another 7 studies in which a variety of attentional tasks were used did not find significant differences in attention during CCR compared with ad libitum eating.^49–53^

One study demonstrated a decrease in attention during CCR^34^; however, attention was reported to improve for participants in 2 other studies.^54^^,^^55^ Participants who restricted their energy intake over 12 weeks while assigned to either a low-fat or Mediterranean diet performed significantly worse on the Bakan vigilance task of sustained attention when compared with those in a control group.^34^ Interestingly, the impairment was greatest among those with the largest decrease in cholesterol level, suggesting a possible mechanism for worse attention.

Attention improved in a study in which Weschler’s WAIS III digit symbol coding subtest^56^ was used by women with overweight who reduced their calorie intake by 20% over 12 weeks.^55^ However, this increase was also apparent in the control group, suggesting practice effects may have been responsible. Siervo et al^54^ used the Trail Making Tests (parts A and B) to test attention. The Trail Making Tests require participants to join consecutive sequential numbers (Part A; 1, 2, 3, and so on) and consecutive but alternating numbers and letters (Part B; eg, 1, A, 2, B). The Trail Making Tests are widely used to measure several cognitive domains, including problem solving and psychomotor speed as well as attention. Results from these tasks showed that participants who restricted calories by 40% over 16 weeks performed significantly better than they did prior to restriction.^54^

#### Fasting studies

Seven studies investigated the effects of fasting on attention. Two studies reported an improvement,^2^^,^^57^ 1 study reported worsening,^58^ and 4 reported no changes.^4^^,^^59–61^ Attention was poorer on an identification task to measure attention after a 12-hour fast; participants’ performance on the task was measured at 0900 and 1600 on separate days.^57^ There was an interesting time-of-day effect, with faster reaction times recorded at 0900 in fasting participants when compared with the nonfasting period. Tian et al^57^ modeled their study on Ramadan fasting, which involves 28 consecutive days of daytime fasting lasting approximately 14 hours (depending on the times of sunrise and sunset), bookended by a prefast meal (sahur) and postfast meal (iftar). Ramadan fasting is particularly strict, with individuals often abstaining from water as well as food. Three included studies examined this type of fasting.^57^^,^^62^^,^^63^ The results of Tian et al^57^ were echoed in a study of cyclists^62^ who underwent Ramadan fasting and showed improved attention. The participants were more accurate on a Rapid Visual Information Processing test during Ramadan, as compared with after Ramadan, further supporting the hypothesis that fasting may increase attention. It is notable that these studies did not account for the effect of lowered blood glucose levels, which would almost certainly decline later in the day in fasted individuals.

Teong et al^2^ performed 1 of only 3 studies that directly compared the effects of CCR and fasting on cognition. They randomly assigned 46 women with overweight or obesity to CCR or IF diets at 70% of energy requirements for 8 weeks. Both CCR and IF groups had improved scores on the Digit Symbol Substitution Test, with no group differences. Green et al^60^ used a modification of the Eriksen and Eriksen^64^ procedure to measure attention and reported no differences between fasted and nonfasted groups. Similarly, Owen et al,^61^ using the Stroop Task, found no significant changes in selective attention after fasting.

Zajac et al^4^ allocated participants to a total fast (0 kcal), “extended distribution modified fasting” (ie, 3 small meals distributed across the day; 522 kcal total), or a bulking condition (not considered in our review) over 7.5 hours on a single day. For the purposes of this review, we treat the total fast condition as fasting, and “extended distribution” as CCR, per our definition of CCR as a deliberate reduction in energy intake below 100% (i.e. studies where food intake is reduced but some food is still allowed). Participants had a 7-day washout period between conditions and were assessed on attention, using the Mackworth Clock Task, which requires participants to press the spacebar on a keyboard as quickly as possible when a probe skips an additional point while travelling a circle’s circumference. There were no differences in scores between fasting and CCR groups, although participants reported more positive, subjective experiences of the extended distribution version of CCR (measured through hunger and food cravings) than with the fasting condition, which may have implications for compliance.

Anton et al^59^ found no significant change in attention for adults with overweight who fasted for 16 h/d for 4 weeks. The Montreal Cognitive Assessment (MoCA) was used to assess attention and concentration. The MoCA was also administered in a recent study of 16 young, healthy adults,^58^ half of whom fasted for 5 days while the others fasted on alternate days. Fasting participants were only allowed water. Although participants undergoing both types of fasting had a decrease in total MoCA scores (indicating worse performance) with a large effect size, this was not statistically significant.

### Inhibition

Inhibition describes the ability to restrain or curtail a behavior, response, or process.

#### Continuous calorie restriction studies

The 3 studies that measured the impact of CCR on inhibition did so using the Stroop task. The Stroop task is used to measure a person’s ability to suppress an automatic response. Participants are presented with color names printed in various colors. They are required to first read the color names aloud, then to read aloud the color of the ink, requiring inhibition of the automatic response of reading the words.

All studies reported improvements in inhibition. One reported slower reaction time for both the 20% CR group and for control participants,^55^ but this was possibly due to practice effects. Another study showed that those who restricted caloric intake by 25% over 6 months had improved performance.^65^ Wing et al^66^ reported participants had faster performance during the interference stage of the Stroop test after a month of 80% CR.

#### Fasting studies

Of 4 studies of the effect of fasting on inhibition, 3 used the Stroop test and found no effect on inhibition.^61^^,^^67^^,^^68^ Zajac et al^4^ used the Color Multi-Source Interference Test, a cognitive interference measure, and similarly found no differences after fasting.

### Set shifting

Set shifting is the ability to move flexibly between different tasks and adapt to rule change, and is a type of cognitive flexibility. The Wisconsin Card Sorting Test^69^ is commonly used to measure set-shifting abilities. It requires participants to classify cards according to certain criteria (eg, number of shapes on the card, color and shape of the symbols on the card) and to state whether the classification is correct. After 10 cards, the rule changes, thus requiring the participant to recognize and adapt to the rule change.

#### Continuous calorie restriction studies

Studies investigating the impact of CCR and fasting on set shifting have reported mixed results, with 1 study reporting no change and another reporting a worsening. Using the Wisconsin Card Sorting Test, participants who restricted their calorie intake by 25% over 3 months showed no change.^65^ Interestingly, some studies that involved fasting or short-term severe CR reported observing improvements in set shifting,^70^ whereas others have found worse performance.^35^^,^^45^^,^^46^^,^^71^

#### Fasting studies

Four fasting studies reported a worsening in set-shifting abilities, and 1 reported improvements. Solianik and Sujeta^70^ assessed set-shifting abilities of participants following a 2-day fast using the Two-Choice Reaction Time Task. Participants are required to respond rapidly to 1 of 2 stimuli by pressing the left mouse button on a computerized screen each time 1 stimulus appears, or the right mouse button when the other stimulus appears. Their results showed significantly faster responses, suggesting an increase in set-shifting ability after fasting. This study recruited a small sample of amateur weightlifters; hence, the results should be viewed tentatively in the context of the small sample size.

By contrast, using the Two-Choice Reaction Time Task, Solianik et al^35^ found that severe CR for 2 days, which mimics the 5:2 diet (500 kcal/d for women, 600 kcal/d for men), resulted in a significant decrease in set-shifting ability. Although an increase in reaction time was not significant, there was a significant decrease in accuracy. In another study by Solianik et al,^72^ 11 older women with overweight or obesity fasted for 48 hours, after which the authors found a significant decrease in set-shifting ability in these women, as compared with controls, also using the Two-Choice Reaction Time Task. These results suggest that the degree of energy restriction may play an important role in the impact on cognitive flexibility, although the sample sizes for these studies limit the strength of their findings.

Using a novel, computerized rule-change task to measure set shifting, Pender et al^46^ used a within-participant repeated measures design to compare individuals when satiated and when fasted for 18 hours. Participants were presented with up to 6 identical nonfood images on a screen. They were required to respond to 1 of 4 questions using Yes or No response keys. Participants were asked to judge if the pictures had an odd, even, high (≥4), or low (≤3) number of pictures. The questions were switched periodically for one-third of each trial. The study authors reported there was a greater cost of switching rules (slower responding) in the fasting condition when compared with the satiated condition, suggesting short-term food deprivation significantly impairs set-shifting ability.

This was a replication of an earlier study that used the same experimental design but presented pictures of a mix of foodstuffs or inedible items. For this study, set-shifting costs significantly increased after fasting, regardless of stimulus type.^45^ Similar results were found by Howard et al^71^: female student participants committed significantly more errors in an adapted affective shifting task (including pictures of food or household items) when they fasted for 20 hours.

### Processing speed

Processing speed refers to the ability to make sense of and respond to information within a particular time frame. Processing speed requires an element of attention and is typically measured using reaction time.

#### Continuous calorie restriction studies

Findings of studies measuring reaction time during energy restriction were mixed, with 4 reporting no change in reaction time,^2^^,^^50^^,^^53^^,^^73^ 2 reporting slower reaction time,^74^^,^^75^ and 1 reporting faster reaction time.^51^

Buffenstein et al^75^ studied 9 women with overweight who were restricted to 800 kcal/day for 1 month. The participants undertook a reaction time test that involved tapping 1 of 5 brass discs, each corresponding to 1 of 5 small red lights. Participants were required to tap the correct disc when a corresponding light was illuminated. All participants were tested first prior to CR and then a second time after 4 weeks of restriction. The authors reported significantly faster mean reaction times and greater accuracy after CR. Given the lack of a control group in this study, it is, again, important to consider the possibility that these results may reflect practice effects.

Halyburton et al^74^ included 93 obese participants during an 8-week clinical trial. They used an inspection time test that required participants to identify the shorter of 2 lines presented together in an image. Those following a diet in which energy intake was reduced by 30% were significantly quicker to identify the target stimulus than control participants who ate normally.

The only study of CCR that reported impairment in processing speed used a computerized finger-tapping task to measure the reaction time of 14 women who restricted energy intake to 50% over 4 months.^51^ Compared with the control group, their reaction time was significantly slower, and, interestingly, it continued to slow (by 10%) after a 3-week weight-stabilization period.

#### Fasting studies

From the fasting literature, 2 studies measured processing speed^57^^,^^67^ and reported mixed results. One used the Staged Information Processing Speed test, which comprises multilevel arithmetic problems the participant is required to solve.^67^ As the levels progress in difficulty, participants are required to respond using the left and right mouse buttons to indicate whether the result is greater than, equal to, or less than 4. Using a within-participant design, 46 university students were tested on fasting (12–16 hours) and nonfasting days over an average of 35 days. The researchers found that reaction time was more impaired on fasting days for participants during medium-difficulty tasks than for lower or higher rated tasks. The authors suggested their findings highlight the importance of considering the role played by task demands and their level of difficulty when comparing studies that attempt to measure a particular cognitive domain after calorie reduction.

Conversely, processing speed improved for a sample of male athletes who fasted for 12 hours. Tian et al^57^ used a detection task, which formed part of a cognitive battery. The detection task uses playing cards on a computerized screen, all of which are red or black jokers. Participants are required to press a key as soon as the center card on screen is turned. Significantly faster reaction times were reported after 12 hours of fasting. Finally, in the study by Teong et al,^2^ which included the Digit Symbol Substitution Test, also measured processing speed, but the authors found that neither CCR nor IF affected processing speed.

### Working memory

WM describes a cognitive function that allows short-term information to be held temporarily for processing.

#### Continuous calorie restriction studies

The impact on WM of CCR was mixed, with 4 studies reporting improvements^32^^,^^71^^,^^72^^,^^74^ and 4 others reporting no changes.^45^^,^^46^^,^^50^^,^^52^ Several studies used a digit-span task. Digit span is often used as a measure of short-term numerical memory. The task requires the participants to recall a series of numbers in the correct order immediately after the numbers are presented. Digit-span backward requires the participant to recall the numbers presented but in reverse order.

One study measured digit span recall by participants who were either in a low-fat or low-carbohydrate foods group, both of which had their energy restricted by 30%.^74^ The authors found that digit-span backward was significantly improved in both groups after 8 weeks of CCR, but a lack of a control group increases the possibility that any change was due to practice effects. In a follow-up, Brinkworth et al^73^ found that after 1 year, the low-carbohydrate and low-fat groups both maintained improvements in the backward digit-span task. They concluded that numerical WM improved after CCR, and that this improvement was likely due to energy restriction alone, rather than dietary change in carbohydrate or fat content.

Bryan and Tiggernann,^55^ who also used the backward digit-span task in their study, found no significant change in numerical WM for those on CCR diets with 20% restriction. Similarly, no change was reported in numerical WM when the digit-span task was performed by elderly participants who restricted their calorie intake by 30% over 3 months.^49^ These findings were echoed in another study that also used the digit-span task to measure performance by obese adults with CCR of 30% over 6 months.^53^

Martin et al^48^ measured non-numerical WM using the Rey Auditory and Verbal Learning Test.^76^ Participants who had their energy intake restricted by 25% improved in WM after 3 months, but not after 6 months. The small effect size (generalized η^2^ = 0.07) led the authors to conclude that practice effects were likely responsible for any change over time (although this does not explain why there was no further improvement at 6 months). Improvements were also found in visuospatial WM in a study in which participants’ performance was measured using a matching-to-sample test. The test requires participants to remember a pattern presented on screen. After 2 seconds, the pattern disappears and is replaced by 2 other patterns side by side. The participant must choose which pattern matches the original. Response time and accuracy both improved after a 75% reduction in calorie intake.^35^

One study, a part of the larger multicenter CALERIE trial, used part of the Cambridge Neuropsychological Test Automated Battery to assess WM of healthy adults who restricted calorie intake by 25% for 2 years.^77^ WM improved significantly in the CCR group, as compared with an ad libitum diet at 24 months, but, interestingly, no significant difference was found between the groups at 12 months. The longer timeline of this study underlines the importance of considering the possibility that changes in caloric intake may induce transitional phases of higher cognitive load and/or deficiencies, followed by a potential for improved performance in certain cognitive domains with time.

#### Fasting studies

Fasting studies reported little change in WM, with 9 studies reporting no change, and 1 study reporting worsening of WM. For instance, Benton and Parker^78^ reported that participants who missed their first meal performed worse on a trigram memory test (recalling a sequence of 3 letters after counting backward by 3 for an allotted time) than those who had either eaten breakfast or consumed a drink containing 50 g of glucose, but differences were not significant.

Harder-Lauridsen et al^63^ also used the Rey Auditory and Verbal Learning Test to measure WM of participants who fasted, using the Ramadan model. No significant changes were found after fasting. However, it is notable this was a small, nonrandomized study of 10 healthy men younger than 35 years. Thus, future studies working within this model would improve upon the validity and generalizability of this test by increasing sample size and varying age and sex. Another study that recruited a small sample of healthy male participants found no significant effect of fasting on WM.^57^

Six other studies reported no changes in WM.^52^^,^^59–61^^,^^70^^,^^72^ The Bakan Vigilance Task was used to measure WM in a sample of 21 female participants who had fasted for up to 24 hours prior to testing.^60^ The task requires the participant to press a key when they detect a string of numbers in a sequence of either 3 odd or 3 even numbers. Up to 9 numbers are presented on a computer screen in a continuous stream and the participant should respond as quickly as possible to the tasks. The number of correct hits were recorded, and no differences were observed between fasted and nonfasted participants. A more recent study by Solianik et al^72^ used a memory search test to assess verbal WM and, again, there were no differences between women with overweight and obesity who had fasted for 48 hours and control participants.

The Rapid Visual Information Processing task, which closely resembles the Bakan Vigilance Task, was used to measure participants’ performance immediately after they had missed 1 meal.^52^ No significant differences in WM were found between fasted and nonfasted conditions. Moreover, 1 of only 3 studies to directly compare CCR and fasting found a significant deterioration in recognition memory (the ability to recognize an item that has been encountered previously) in individuals with central obesity eating 5:2 pattern diets and who had fasted overnight (at least 12 hours).^2^ Kim et al^23^ noted that 12 of the participants were older than 60 years, which may have affected their results.

### Psychomotor speed

Psychomotor ability refers to the coordination of cognitive and motor processes in response to environmental cues. Psychomotor speed is the speed at which an individual can output a response to a stimulus and can be calculated using a variety of different movement-based tasks.

#### Continuous calorie restriction studies

Only 1 CCR study measured psychomotor speed and found no changes. The Pursuit Tracking Test was used to measure fine psychomotor speed of 9 obese women during a 2-day CCR that mimicked the popular 5:2 diet, reducing calorie intake by 75%.^35^ The Pursuit Tracking Test requires participants to track a moving box across a screen, ensuring that as the box moves, the mouse cursor remains inside the box. The study observed no effects on performance after 2 consecutive CCR days.

#### Fasting studies

Three fasting studies measured psychomotor speed and all reported a worsening in this domain. Psychomotor speed was measured after a 16-hour fast, using both the Catch Game task and a tapping task.^67^ The Catch Game tests for hand–eye coordination and response speed. It requires participants to move a “paddle” at the bottom of a screen so it can catch objects that are falling. No differences were observed between fasting and nonfasting days in the tapping task; however, the time until first move was significantly longer on fasting days for the Catch Game task.

Another study that used a tapping task found psychomotor speed decreased in participants after a 24-hour fasting period compared with 2 other participant groups that had missed either 1 or 2 meals prior to testing.^60^ Using a sample of 21 healthy female participants, the 2-finger tapping task involved the pressing of the “1” and “2” buttons on a keyboard alternately using the first and second fingers of the preferred hand until reaching 300 presses. The outcome was measured by taps per second. There was a significant effect of food deprivation on tapping rate. In another study by Green et al^52^ in which the same task was used, the authors also reported the same effect after short-term food deprivation (missing 1 meal prior to testing when compared with a satiated condition).

## DISCUSSION

Of the 34 studies reviewed, 24 reported finding significant changes in cognition. Overall, CCR studies were more likely to report cognitive improvements, whereas deficits were more likely to be reported in fasting studies, suggesting that the degree and duration of CR may play important roles in affecting the direction of any impact on cognition. It is possible that a mild restriction of calorie intake is associated with improvements in some cognitive domains, whereas severe restriction or total fasting for long periods is more likely to lead to worsening performance.

Most impairments were observed in the domains of set shifting and psychomotor speed, across both fasting and CCR studies. Set-shifting ability decreased after a 20-hour fast and 48-hour fast, respectively, in 2 studies^71^^,^^72^ and 1 study from the literature on CCR reported impairments after severe CR (75%) over 2 days.^35^ This most closely resembles the 5:2 IF diet in which calorie intake is restricted by 75% for 2 days each week. The only study to find a significant difference between fasting and CCR (on WM, in this case) also mimicked a 5:2 diet.^2^ This suggests that the degree of CR may play an important role in changes to set-shifting and WM ability during dieting.

Of the 3 fasting studies that measured psychomotor speed, all reported significant impairments. Notably, these studies recruited healthy university students and their findings were similar to studies of young people with AN.^79^ Therefore, more research investigating the effect on psychomotor speed after engaging in restrictive eating in the form of fasting would add to our understanding of a potential cognitive profile for EDs.

Most improvements were observed in the domains of inhibition, WM, and attention (in 9 studies). The majority of these studies examined CCR, although surprisingly, 2 fasting studies reported an improvement in attention.^57^^,^^62^ Finally, there were inconsistent findings from the literature regarding the impact of CCR and fasting on processing speed.

### Limitations

Variability in methodology makes it difficult to directly compare the results of the studies for any of the cognitive domains. For instance, whereas some used a combination of specific tasks to measure cognition,^60^ others used more extensive cognitive batteries.^67^ Furthermore, cognitive domains were measured in numerous ways. For example, processing speed was measured differently in 2 of the studies with contrasting results.^57^^,^^67^ Finally, the studies reviewed contain a mix of within-participant and between-participant designs.

Another area of variability in study design is the duration of fasting. Whereas some studies measured cognitive outcomes within short time frames (4 hours of fasting^52^), others included much longer fasts (5 days^58^). This limits our ability to make conclusions across studies about the effects of fasting on cognitive function. However, it seems likely that during short periods of fasting, cognitive performance is preserved or even improved, whereas longer periods of fasting may lead to negative impacts.

Few studies accounted for major confounders such as exercise, diet, and time of day. For instance, 3 of the studies we reviewed found that performance improved in the afternoon.^57^^,^^62^^,^^67^ In addition, research indicates a close link among dietary intake, exercise, and cognition.^80^ Some studies asked participants to maintain their level of exercise, controlled for physical activity, and/or did not account for exercise at all, but some did include light exercise conditions that were standardized across interventions.^48^^,^^51^^,^^62^ Moreover, despite the widely accepted effect of glucose on cognition, fewer than half of the studies reviewed here took this into consideration.

Of particular note is the role of confounding variables in Ramadan studies, where there was variability in the time of day participants started and ended their fasts and, because participants may need to get up before sunrise to prepare food, sleep disturbances were common.^62^ Indeed, although there are methodological similarities between the Ramadan fasting studies themselves,^57^^,^^62^^,^^63^ such as all including before, during and after Ramadan testing points, and it is important to consider this type of fasting as a common, religiously motivated type within healthy adults, this model of fasting does introduce differences from the other studies in this review.

The majority of these studies were laboratory based. Under these conditions, motivation is likely to be extrinsic (ie, it is likely that participants are motivated by outside sources such as financial gain). Given that diets are costly, most dieters are intrinsically motivated, and intrinsic motivation is usually more successful in changing long-term dieting behavior.^81^

Lastly, in this literature review, we focused on experimental studies that manipulated total calorie intake; search terms did not include an exhaustive range of cognitive processes. The search parameters were limited to focus on domains that might also inform an eating disorder profile; therefore, other domains that might be affected by a reduction in calorie intake were not included.

## CONCLUSION

In this review, we identified 33 studies of CCR and fasting in humans, with 23 demonstrating significant changes in cognition. Despite variation across the domains, the results suggest that CCR may benefit inhibition, processing speed, and WM, and worsen cognitive flexibility. The results of fasting studies suggest that fasting is associated with impairments in cognitive flexibility^42^^,^^43^^,^^69^^,^^70^ and psychomotor speed.^49^^,^^57^^,^^65^ Measures of attention and processing speed reported varied outcomes for fasting studies, whereas inhibition and WM appeared unaffected. Inconsistent findings were reported in all cognitive domains across the studies, likely due to variations in fast duration and tasks used.

Overall, it appears that the length of fasting and/or the degree of energy reduction (eg, 50% through CCR, as opposed to 100% through fasting) are both likely to be important factors affecting cognitive function. Research could further elucidate this by testing participants undergoing various degrees of restriction over a range of periods. Vitousek et al’s^82^^,^^83^ animal research may provide a useful lens for interpretation of the literature. Their review suggests there is a threshold up to which a reduction of food intake can benefit cognition, after which there may be detrimental impacts on cognitive functioning as well as physical health. This has important potential implications for restrictive EDs in which CR is both extreme and sustained, and diets that involve IF, where the goal of health optimization must be considered in conjunction with the fine line between optimal and suboptimal CR.

### Future directions

Moving forward, an emphasis on reversal design to assess participants’ cognitive abilities before, during, and after dietary intervention may be of benefit. Randomizing the order in which participants perform under fasting and nonfasting conditions, as well as the use of a wider variety of cognitive tests, to reduce practice effects, may also strengthen methodological designs. Future research should explore fasting for a range of durations to determine tipping points when benefits may reduce and costs increase. Given that exercise is a core feature of many weight-loss programs, a prominent characteristic in EDs, and has clear associations with cognition,^84^ researchers should aim to carefully measure exercise or incorporate exercise interventions into their study design, as well as account for other factors such as stress and sleep patterns. Future work should also account for blood glucose and insulin levels as potential confounding factors when measuring changes in cognition. Finally, studies that recruit participants who are already dieting outside of laboratory conditions will likely have higher external validity and reduced dropout rates while increasing ecological validity.
